# Correlation between TMD and Cervical Spine Pain and Mobility: Is the Whole Body Balance TMJ Related?

**DOI:** 10.1155/2014/582414

**Published:** 2014-06-19

**Authors:** Karolina Walczyńska-Dragon, Stefan Baron, Aleksandra Nitecka-Buchta, Ewaryst Tkacz

**Affiliations:** ^1^Department of Temporomandibular Joint Dysfunction and Orthodontics, Medical University of Silesia, Pl. Traugutta 2, 41-800 Zabrze, Poland; ^2^Institute of Theoretical and Applied Informatics, Polish Academy of Sciences, 5 Batycka Street, 44-100 Gliwice, Poland

## Abstract

Temporomandibular dysfunction (TMD) is considered to be associated with imbalance of the whole body. This study aimed to evaluate the influence of TMD therapy on cervical spine range of movement (ROM) and reduction of spinal pain. The study group consisted of 60 patients with TMD, cervical spine pain, and limited cervical spine range of movements. Subjects were interviewed by a questionnaire about symptoms of TMD and neck pain and had also masticatory motor system physically examined (according to RDC-TMD) and analysed by JMA ultrasound device. The cervical spine motion was analysed using an MCS device. Subjects were randomly admitted to two groups, treated and control. Patients from the treated group were treated with an occlusal splint. Patients from control group were ordered to self-control parafunctional habits. Subsequent examinations were planned in both groups 3 weeks and 3 months after treatment was introduced. The results of tests performed 3 months after the beginning of occlusal splint therapy showed a significant improvement in TMJ function (*P* > 0.05), cervical spine ROM, and a reduction of spinal pain. The conclusion is that there is a significant association between TMD treatment and reduction of cervical spine pain, as far as improvement of cervical spine mobility.

## 1. Introduction

Recent years have seen a significant increase in the number of patients suffering from temporomandibular disorders (TMD) [1]. According to various sources, 8 out of 10 patients coming to the dentist are found to have bruxism or TMD [2].

The issue of relationships between temporomandibular disorders and body posture is still a source of speculations. The knowledge about connections between distant body districts has to be proven by appropriate diagnostic procedures and instruments.

TMD are musculoskeletal disorders needing a multidisciplinary effort to manage with other professionals (e.g., neurologist, laryngologist, and psychiatrist) [3].

Because of the variety of TMD symptoms, many patients had a history of multiple treatments and medications and were treated previously by laryngologists, neurologists, or physiotherapist, but the therapy did not bring the expected, long lasting results. According to currently prevailing theories, temporomandibular dysfunction is considered to be associated with imbalance of the whole body [4].

In addition, the body as a whole operates on the principle of compensation, when it comes to disturbances in the upper quarter, such as increased muscle tension; this will lead to compensatory changes within the muscle tension in the spinal region so as to force the correct position/posture. These adaptive changes occur at all levels, within tolerance of the body [5, 6].

When the body capacity to compensate for the pathological changes progressing in given areas is exceeded, however, imbalance sets in and pathological symptoms will appear. Each individual, obviously, has a unique compensation limit beyond which such symptoms are triggered off.

It was pointed out by many authors that pain in the upper quarter and masticatory motor system may be caused by cervical spine disorders (generally by dysfunction of muscular origin) and vice versa [7–9].

It could be explained by specific functional and morphological connections between the cervical and temporomandibular regions.

## 2. Materials and Methods

The sample was comprised of 60 individual (30 female, 30 male, age 18–40) and was divided into two groups with randomization. Study and control groups both consisted of 30 people with TMD, cervical spine pain, and limited cervical spine range of movements (ROM). Subjects were directed from Cooperating Orthopaedic Service.

Groups were not different regarding age and gender.

Patients from both groups met the criteria for inclusion and exclusion of studies (Table 1).

Patients from both groups were recruited from cooperating clinics and previously diagnosed by an orthopaedist who excluded morphological and degenerative changes of cervical spine. Cervical spine pain was diagnosed by an orthopaedist according to the Neck Pain Task Force recommendations [10].

Each patient had to have had cervical spine pain for at least 12 months in multiple episodes at a frequency of at least once a week. Patients were included in the study if having pain in the area between occiput and C7.

Subjects gave written consent to participate in the study. The study was approved by the Ethics Committee of the Medical University of Silesia (number KNW/0022/KB1/6/I/10 from 16.03.2010).

Each patient was examined three times. At the 3-week and 3-month evaluations, symptoms of TMD and cervical spine pain and mobility were studied.

The examination included the following:

1. medical history and physical examination, based on a survey card (according to RDC/TMD);
2. analysis of pain, using the visual analogue scale (VAS) and the cervical Oswestry scale for the cervical spine;
3. TMJ functional evaluation by JMA device;
4. cervical spine motion evaluation with the MCS device.

In order to describe individual TMD symptoms, the entire sample filled out a questionnaire according to research diagnostic criteria for TMD, the translated Polish version (RDC/TMD axis I). The questionnaire focuses on symptoms specifically in the jaw-face, neck, shoulder girdle, intensity of spinal pain, and any other complaints of TMD and spinal origin. Presence of symptoms was marked according to duration, frequency, and intensity. The survey card was completed by each patient 3 times during three consecutive examinations which enabled a comparison of symptoms between groups according to treatment provided in treated group.

On a questionnaire, patients indicated the intensity of spinal pain experienced at the time of examination on a 100 mm visual analogue scale (VAS). Additionally, subjects described symptoms of pain and reduced mobility of cervical spine by filling in the cervical Oswestry scale.

Clinical examination was performed according to RDC/TMD guidelines, too.

Previously trained examiner assessed face symmetry, dentition, and occlusion, as far as “upper quarter” muscle tenderness to palpation with an emphasis on the masticatory muscles, trapezius muscles, suprahyoid muscles, infrahyoid muscles, sternocleidomastoid muscles, and neck muscles in the region of the linea nuchae. Each time the muscle tension was examined by the same examiner.

Mandibular motion was recorded using jaw motion analyzer (JMA) from Zebris, (GmBbH) and the software provided (WinJaw) [11]. The device allows recording mandibular position and movements.

The subjects were provided with an explanation as to the objective of the axiographic examination and its course as well as what types of mandibular movements should be made and how. For each examination, it was necessary to make a paraocclusal clutch mounted on the vestibular surface of the lower teeth and fitted with an electronic sensor. The tool was made of light-cured Multitray (Espe).

**Figure 1:**
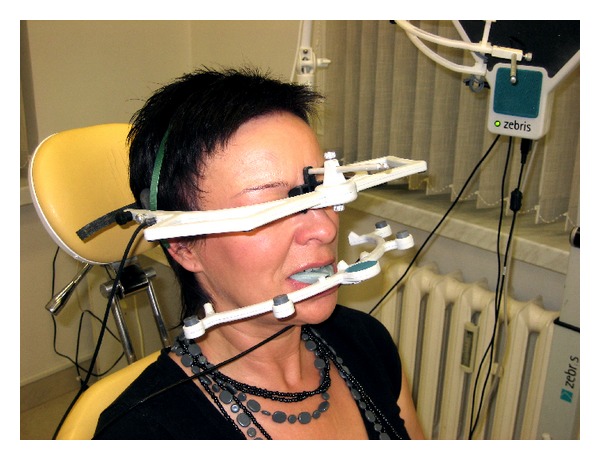
Patient during the mandibular movements' examination (JMA).

The study was based on the performance of patients' movements: opening and closing of the mandible, lateral movements, protrusion, and retrusion. To avoid bias, all subjects performed each trials three times. For each movement, the baseline position was the mandibular rest position. It seems that the rest position should be the starting point when assessing the motor function of the stomatognathic system using instrumental techniques (Figure 1).

The advantages of this system are the ease of use and a positional accuracy of about 100 micrometers. Software allowed creating data report, which consists of graphic diagrams of TMJ function (e.g., Condyle path, maximal opening, and Bennett angle).

**Figure 2:**
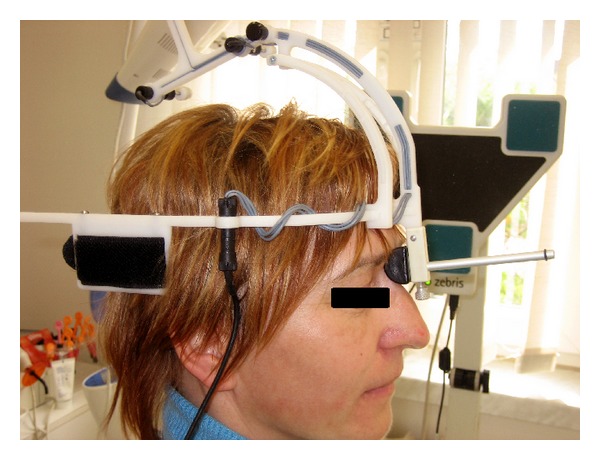
Patient during the cervical spine movements' examination.

Afterwards, the MCS (Zebris, GmbH) ultrasonic-based device was used to collect external kinematic data of the cervical spine movements. Patients with a neutral (comfortably seated) position performed maximal head movements: flexion, extension, rotation to the right and left side, and lateral flexion movements. Each movement was repeated three times in order to minimise measurement errors. The system was calibrated before each measurement. Data were monitored in a real time (Figure 2).

Thanks to the repeatable measurements, values were found describing the cervical spine ROMs, presented in the form of relevant graphs.

After the subjects' examination, data containing information about the quality and range of movement in both the TMJ and cervical spine were stored on a personal computer.

**Figure 3:**
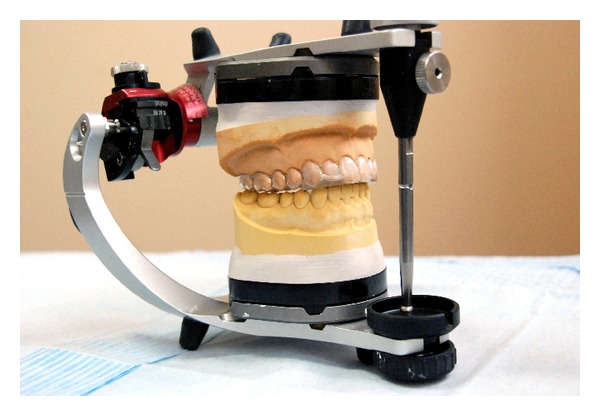
SVED appliance.

After the first examination, each patient selected for the treated (experimental) group was supplied with an occlusal splint. Every patient suffered from TMD of muscular origin (RDC/TMD axis I); therefore, subjects were supplied with an occlusal splint SVED (Sagittal Vertical Extrusion Device). SVED is a removable, flat-plane appliance which makes contact only with the anterior teeth in the opposing arch [12]. It disengages the posterior teeth and thus eliminates their influence in the function of the masticatory system by changing the input signal from proprioceptive fibres contained in the periodontal ligament of the posterior teeth (Figure 3).

The SVED appliance is used in case of hyperactivity of masticatory muscles, without the occlusal reason of TMD [13]. It is usually used to promote jaw muscle relaxation in patients with stress related pain symptoms like headache or neck pain of muscular origin. The splint also obliges the patient to find a new mandibular position, which results in a muscular balance. Patients were ordered to wear the occlusal splint during sleep, but not more than 8–10 hours per day.

According to many researchers, there is no ideal way to handle the problem of control treatment, especially in splint studies. The use of a placebo control group can balance the nonspecific effects in the treatment group and allow for independent assessment of the real treatment effect [14]. In our study control, subjects were instructed to self-control clenching and other parafunctional habits.

The statistical analysis of the results was performed using the statistical package STATISTICA 9.0 (StatSoft). The test probability of *P* < 0.05 was assumed to be significant while the test probability of *P* < 0.0001 was highly significant.

## 3. Results

60 subjects were examined: 30 belonged to the treated group and 30 to the control group. Patients were randomly admitted to groups. The characteristics of age and gender for both groups are shown in Table 2.

All patients were simultaneously assessed by the same examiner.

### 3.1. RDC/TMD Diagnoses

Referring to TMD research diagnostic criteria, in patients from both treated and control groups myofascial pain (I) or disc displacement with reduction (DDR, IIa) was diagnosed.

After a three-month therapy with an occlusal splint considerable improvements of TMJ function were found in the experimental group, with 78% of the subjects reporting no DDR symptoms or acoustic phenomena like clicks during mandible movements; the abduction path of the mandible was symmetrical, and there was no pain during the movements (Table 3).

Most interestingly, however, there were changes on the condyle path in the TMJ during the measurements made with a JMA. Deviations within the condylar path which had been noticeable in the first examination (such as lack of symmetry between the length of the path in the right and left TMJ) became reduced in 28 subjects as a result of the treatment, and during the third measurement, the graphs of the condylar paths were asymmetrical on both sides in as few as four subjects. In 24 subjects, there were considerable improvements, which also improved the TMJ function.

In the control group, no changes in the TMJ function were observed in the clinical examination or instrumental check with a JMA in successive examinations.

Muscle tension was examined by palpation by the same examiner. In all subjects, upper quarter muscle tenderness (masticatory muscles, semispinalis muscles, trapezius muscles, sternocleidomastoideus muscles, suprahyoid muscles, and neck muscles in the region of the linea nuchae) was diagnosed during three consecutive examinations. The presence of muscle pain and tenderness during palpation was registered in both experimental and control groups.

During the third examination, the muscle tension of the subjects in the experimental group lowered considerably and they reported lack of pain in the examination by palpation. Out of the 27 subjects in whom intensified tension of the examined muscles had been found, 22 reported no complaints during the third examination. No significant changes were found in the control group.

### 3.2. Spinal Pain

**Figure 4:**
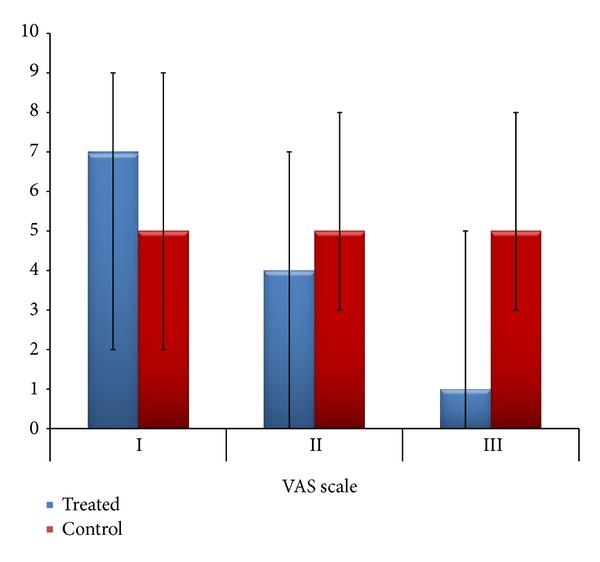
The VAS scale score according to groups during three examinations.

The whole group showed cervical spine pain. Cervical spine pain according to VAS scale in a treated group significantly improved during three-month therapy (Figure 4).

During treatment, cervical spine pain diminished and after 3 weeks it occurred in 39% of subjects and after 3 months pain was only in 8% subjects from treated group (2 subjects).

The difference between treated and control groups was statistically significant (*P* < 0.0001).

### 3.3. Cervical Spine ROM

During the first examination, cervical limited ROM at least during one of the tested movements was reported in 60 subjects.

For each measurement, a relevant physiological standard was established, to which the cervical spine ROM results were referred [15]. The norm assumed was dependent on the gender and age of the subject.

Many authors claim that in the ROM examinations of the cervical spine it is not correct to treat an imposed and inflexible range of values within which the ROM should be included as the only indicator. As the resultant data are dependent on too many additional factors, in the study we placed special emphasis on the comparison between the results from the first, second, and third examinations and on the assessment whether they have been changed or improved, not merely whether they fell within the standard.

After introducing the occlusal splint therapy, cervical spine mobility improved.

**Figure 5:**
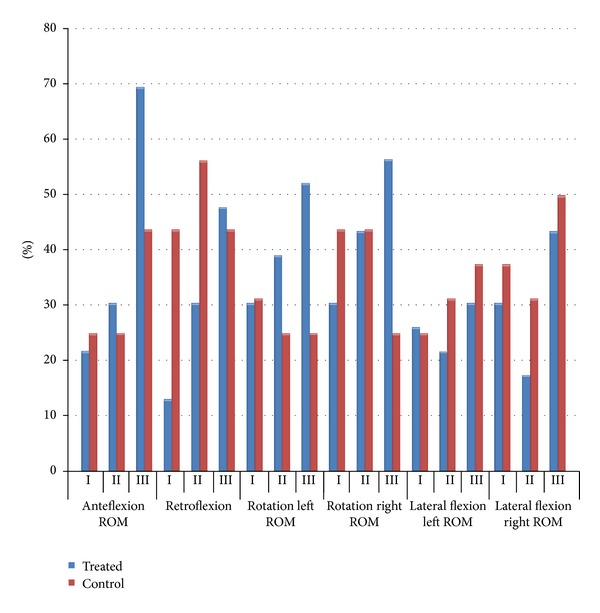
Cervical spine mobility results.

The highest improvement was seen during the flexion movement, which, on the 1st examination only in 22% of patients, was within normative values. During the 3rd examination in 70% of patients from treated group flexion movement conformed the norm (Figure 5).

For the anteflexion movement, the improvement of the results was highly significant (*P* = 0.0006); that is, there were more subjects in the experimental group with the result conforming to the norm.

Likewise, for the retroflexion movement, the results were improved by a highly significant factor (*P* = 0.0082); that is, there were more subjects in the experimental group with the result conforming to the norm.

In the control group, no significant (*P* > 0.05) changes were found; that is, there was no ROM improvement in the cervical spine towards the values in the norm.

The results of improving the mobility and reduction of cervical spine pain influenced the cervical Oswestry scale score. The average score on the first examination in a treated group was 9.22 points and during therapy, after 3 months, the average score changed to 3.71.

## 4. Discussion

The results obtained have confirmed a correlation between the pathologies and the positive impact of treatment within the motor aspect of the stomatognathic system on the alleviation of spine pain, even in subjects experiencing such pain for many years.

It is important to understand the complex interrelations between the stomatognathic system and pain and dysfunctions in other areas of the body in order to be able to treat patients more efficiently and effectively at the initial stage, when painful symptoms appear and when curing them is possible as well as much swifter and more efficient. To be able to make successful therapeutic interventions, dental surgeons should cooperate in an interdisciplinary fashion with neurologists, orthopaedists, or laryngologists. They all should also take such interdependencies into account in their diagnostic work with their own patients.

Scientists often note the importance of a holistic approach to therapy. There are many voices in favor of this approach that symptoms of the disorder are usually not isolated and the dysfunction of one region of the body also applies to other regions [16–20].

Although the etiology of cervical spine pain very often remains unexplained, medical specialists in many cases report the comorbidity of dysfunctions in the stomatognathic system and the pain syndrome in the cervical spine [4]. Numerous scientific reports confirm that many researchers have embarked on the examination of the impact of disorders in the “upper quarter” on body posture and pain experienced in various areas of the body [21]. In studies conducted thus far, however, the focus has been mainly to prove the presence or absence of dependence between dysfunction of the stomatognathic system and pain in the cervical spine. The most commonly applied methodology was questionnaires with questions concerning cervical spine pain and complaints of the motor aspect of the stomatognathic system [22]. On that basis, researchers would look for a link between the dysfunction in the motor aspect of the stomatognathic system and the pain felt in the cervical spine. Our study, however, included a therapy with an occlusal appliance, with no other invasive treatment methods used. By applying treatment with an occlusal splint in the experimental group, a vast majority of the subjects reported improvements and the total disappearance or considerable alleviation of cervical spine pain and TMD symptoms, while the mobility of the cervical spine improved considerably as well.

In case of TMD, there are often large discrepancies between therapists concerning type of occlusal splint most appropriate to use. Many types of splints can be distinguished, for example, stabilization splint, repositioning splint, relaxation splint, or splints only for protecting oral tissues. SVED splint, which is a typical relaxing appliance, was used because of its influence on jaw muscles. No studies about different types of splints used in patients with both TMD and spinal pain were found [23].

## 5. Conclusions

Our studies as well as the clinical followup suggest that TMD is very frequently present along with pain in the cervical spine. The key aspect of the studies described here is the considerable ROM improvement in the cervical spine and the elimination of cervical spine pain felt there by the subjects in the experimental group. Taking into account the results of our study, it seems obvious that interdisciplinary cooperation between orthopedist, laryngologist, neurologist, and dentist is necessary and essential.

## Figures and Tables

**Table 1:** Inclusion and exclusion criteria of studies.

Inclusion criteria	Exclusion criteria
(1) Spinal pain (2) Women, men (3) Age between 18 and 40 (4) Functional changes of the spine, muscle-related (5) Temporomandibular joint disorder/bruxism (6) Patient agreement	(1) After spine surgery (2) Congenital or degenerative changes of the spine confirmed radiologically (3) Neuropathy (4) Ongoing medication or physiotherapy (5) TMJ internal derangement

**Table 2:** Characterization of the sample according to age and gender.

Gender	Group		Total
	Treated	Controls	
Female	16	14	30
Male	14	16	30
Mean age in years	32,65	34,87	33,76

**Table 3:** Symptoms of DDR and myofascial pain during 3 examinations.

	Myofascial pain (treated/control)		Disc displacement with reduction Left side (treated/control)		Disc displacement with reduction Right side (treated/control)	
Examination 1	27	29	13	11	15	12
Examination 2	19	26	8	11	10	12
Examination 3	4	25	3	10	6	12
